# Histone demethylases in chromatin biology and beyond

**DOI:** 10.15252/embr.201541113

**Published:** 2015-11-12

**Authors:** Emilia Dimitrova, Anne H Turberfield, Robert J Klose

**Affiliations:** ^1^ Department of BiochemistryUniversity of OxfordOxfordUK

**Keywords:** chromatin, demethylase, epigenetics, histone methylation, Chromatin, Epigenetics, Genomics & Functional Genomics, Post-translational Modifications, Proteolysis & Proteomics

## Abstract

Histone methylation plays fundamental roles in regulating chromatin‐based processes. With the discovery of histone demethylases over a decade ago, it is now clear that histone methylation is dynamically regulated to shape the epigenome and regulate important nuclear processes including transcription, cell cycle control and DNA repair. In addition, recent observations suggest that these enzymes could also have functions beyond their originally proposed role as histone demethylases. In this review, we focus on recent advances in our understanding of the molecular mechanisms that underpin the role of histone demethylases in a wide variety of normal cellular processes.

**Glossary**
2‐OG: α‐ketoglutarate
53BP1: 53 binding protein 1
AR: androgen receptor
ARID: AT‐rich interaction domain
ATM: Ataxia‐Telangiectasia mutated kinase
BRCA1: breast cancer type 1 susceptibility protein
BUBR1: BUB1‐related protein kinase
CDK1: cyclin‐dependent kinase 1
CK2: casein kinase 2
c‐Myc: myelocytomatosis oncogene
CoREST: REST corepressor
DNMT1: DNA methyltransferase 1
Epe1: enhancement of position effect 1
ES cell: embryonic stem cell
FAD: flavin adenine dinucleotide
FBXL: F‐box and leucine‐rich repeat protein
HCF‐1: host cell factor 1
HDAC: histone deacetylase complex
HP1γ: heterochromatin protein 1γ
iPS cell: induced pluripotent stem cell
JARID: jumonji domain ARID‐containing protein
JHDM: JmjC domain‐containing histone demethylation protein
JmjC: jumonji C
KDM: histone lysine demethylase
lncRNA: long non‐coding RNA
LSD: lysine specific demethylase
MAD2: mitotic arrest deficient 2
MINA53: Myc‐induced nuclear antigen 53
MLL: mixed‐lineage leukaemia
MRE11: meiotic recombination 11
MRG‐15: MORF‐related gene on chromosome 15
NFATc1: nuclear factor of activated T‐cells, cytoplasmic, calcineurin‐dependent 1
NO66: nucleolar protein 66
NuRD: nucleosome remodelling deacetylase
O‐GlcNAc: O‐linked N‐acetylglucosamine
OGT: O‐GlcNAc transferase
PARP1: poly (ADP‐ribose) polymerase 1
PARylation: poly (ADP‐ribosyl)ation
PCNA: proliferating cell nuclear antigen
PHD: plant homeodomain
PHF: PHD‐finger protein
PKA: protein kinase A
Pol II: polymerase II
PPARγ: peroxisome proliferator‐activated receptor
PRC: polycomb repressive complex
Prm1: protamine 1
RAD51: radiation sensitive mutant 51
RBP2: Retinoblastoma‐binding protein 2
REST: RE1‐silencing transcription factor
RNAi: RNA interference
RNF: Ring‐finger protein
SCF: Skp1‐Cul1‐Fbox complex
SCNT: somatic cell nuclear transfer
SETD2: SET domain‐containing protein 2
SIN3: switch‐independent 3
SMCX: selected mouse cDNA on X
SPT6: suppressor of Ty6
SUMO: small ubiquitin‐like modifier
SVIL: supervillin
SWI/SNF: SWItch/sucrose non‐fermentable
SWIRM: Swi3p, Rsc8p and Moira domain
Tnp1: transition nuclear protein 1
TPR: tetratricopeptide repeat region
TYW5: TRNA‐YW synthesizing protein 5
U2AF65: U2 auxiliary factor 65
UTX: ubiquitously transcribed X‐chromosome tetratricopeptide repeat protein
UTY: ubiquitously transcribed Y‐chromosome tetratricopeptide repeat protein
WD‐40: Tryptophan‐aspartic acid (W‐D) repeat 40
ZF: Zinc‐finger domain

## Introduction

Multicellular organisms require diverse cell types to support the complex yet orchestrated processes inherent to their development, physiology and reproduction. To initiate and maintain functionally diverse cell types, mechanisms to precisely control how cells use their DNA‐encoded information have evolved. This is in part achieved by wrapping DNA around histone proteins to form chromatin, which can regulate how genes are expressed, DNA information is replicated and segregated during cell division, and DNA damage is sensed and repaired.

Through studying the function of chromatin in these fundamental processes, it has become clear that many of its effects are mediated through post‐translational modifications on histone proteins. This is exemplified by methylation, which occurs on numerous lysine and arginine residues in histones and has been intensely studied since enzymes that catalyse these modifications were discovered. Histone lysine methylation (me) can occur in the mono‐ (me1), di‐ (me2), or tri‐methyl (me3) state, while arginine methylation is found in various symmetric and asymmetric mono‐ and dimethylated states (reviewed in 1, 2). In some very specific instances, histone methylation can directly affect chromatin structure. However, it appears that “reader” proteins, which bind specifically to methylated histones and recruit additional activities to drive functional outcomes on surrounding chromatin, are the central determinants underpinning the function of these post‐translational modifications. Reader proteins often have the capacity to recognize defined methylation states, meaning that individual residues can encode different functional outcomes depending on their methylation state 3.

Initially, it was believed that histone methylation may be irreversible, despite early biochemical work suggesting that enzymatic activities that remove these modifications may exist in cells 4, 5. This discrepancy was resolved with the discovery of the histone demethylase KDM1A/LSD1, which actively removes methylation from histone H3 on lysine 4 (H3K4) via the activity of its amine oxidase domain, using FAD as a cofactor 6. Shortly after this important discovery, KDM2A/JHDM1A/FBXL11 was shown to demethylate H3K36 via its JmjC domain, which coordinates iron to mediate a 2‐OG‐dependent demethylation reaction 7. Since these initial discoveries, an extended family of related demethylase enzymes has been identified and their substrate specificities have been characterized in detail (reviewed in 8, 9, 10). This has revealed that most of the abundantly methylated lysine residues in histones have a corresponding demethylase enzyme. In addition, it has been proposed that a JmjC domain‐containing protein, JMJD6, may function as an arginine‐specific histone demethylase 11, 12; however, the precise nature and biological relevance of this reaction remains a matter of dispute 13, 14, 15. The study of histone lysine demethylase enzymes over the past decade has revealed that dynamic regulation of histone methylation plays central roles in fundamental chromatin‐based processes. Importantly, misregulation of histone demethylases has been implicated in a wide range of human disorders, including cancer. Based on these observations, histone demethylases are now emerging as central therapeutic targets for small‐molecule‐based inhibition 16.

A series of excellent reviews have provided an extensive and detailed examination of individual histone demethylases, the biochemical characterization of their substrates and the general roles these factors play in development and disease biology 8, 9, 17, 18. Here, we will instead focus on a series of new discoveries that together are beginning to illuminate some of the more general yet fundamental molecular principles that guide how demethylases recognize their appropriate substrates, control gene expression in cell fate transitions and protect genomic integrity in normal cells.

## Recognizing chromatin substrates, targeting histone demethylases and regulating their activity

**Figure 1:**
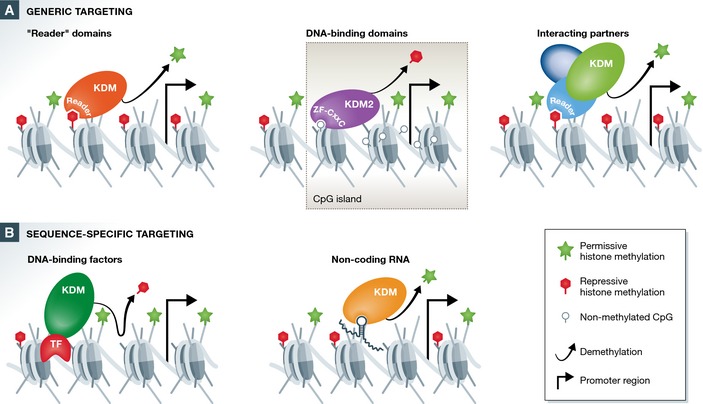
Mechanisms regulating targeting and occupancy of histone demethylases on chromatin (A) Generic targeting mechanisms. Many histone demethylases encode “reader domains”, including PHD, Tudor and TPR domains (left), that bind and read histone modifications found broadly throughout the genome. These interactions function to target histone demethylases to chromatin and regulate their activity. Some histone demethylases interact with chromatin via direct binding to DNA. This is exemplified by the KDM2 histone demethylases that are targeted generically to CpG islands, resulting in localized removal of histone methylation at these sites (middle). Histone demethylases are often found in large multi‐protein complexes, which contain other chromatin‐binding proteins that function to target these enzymes to chromatin (right). (B) Sequence‐specific targeting mechanisms. Histone demethylases in some instances are targeted to specific sites in the genome through interaction with transcription factors (left) or with lncRNAs (right).

Genome‐wide mapping of histone lysine methylation in cells has demonstrated that these modifications are often restricted to, or absent from, very specific regions of the genome 19. The establishment of these profiles relies on the regulated activity of histone methyltransferases and demethylase enzymes. Although we have detailed information about how the active site defines histone residue specificity for these enzymes 20, 21, 22, 23, 24, 25, 26, additional and more complex targeting and regulatory mechanisms must underpin the genome‐wide methylation patterns observed *in vivo*. Recently, it has become clear that non‐catalytic domains within histone demethylases and interactions with other proteins are key determinants in controlling chromatin targeting and catalytic activities. This has revealed that a series of generic and sequence‐specific targeting mechanisms determine the binding and activity of these factors on chromatin (Fig 1).

### Beyond the active site

Histone lysine demethylases are often large multi‐domain proteins, suggesting that these additional domains may play a role in their targeting, substrate selection and activity. Indeed, very early structural studies on KDM1A revealed that its SWIRM and oxidase domains directly interact, anchoring the active site to the histone tail to support catalysis 27, 28, 29, 30, 31, 32. Interestingly, although KDM1B/LSD2 has a very similar domain architecture to KDM1A, it contains a unique linker region within its SWIRM domain that binds to a region of the H3 tail away from its H3K4 substrate lysine 33. As a result, KMD1B binds to a much longer sequence of histone H3 than KDM1A, and its activity requires engagement with both substrate and non‐substrate regions of the histone tail through different protein domains. KDM1B also contains a unique N‐terminal Zn‐finger domain that physically interacts with its SWIRM domain and is essential for cofactor binding and catalytic activity 33, 34. Together, this exemplifies how histone demethylase activity is not only defined by the active site itself, but also requires complex interactions between the substrate and addition domains.

### Instruction from histones

Interestingly, many of the non‐catalytic protein domains found in histone lysine demethylases encode “reader” domains, such as TPR, Tudor and PHD domains, that bind to histones and recognize post‐translational modifications (Fig 1A) 8, 35, 36. For example, KDM7 demethylases encode a PHD domain that binds to H3K4me2/3 and recruits these enzymes to regions of the genome enriched for this modification 37, 38, 39, 40, 41, 42. Reader domain interactions can also have functions beyond simple targeting, as KDM7B/PHF8/JHDM1F binding to H3K4me3 via its PHD domain leads to an allosteric activation of its demethylase activity, stimulating the removal of transcriptionally repressive H3K9me2 and H4K20me1 modifications 37, 39. H3K4me3 is often associated with transcriptionally permissive or active regions of chromatin, suggesting that recruitment and activation of KDM7B at these sites may limit repressive histone modifications from spreading into these regions 37, 38, 39, 43, 44. In keeping with this possibility, loss of KDM7B results in abnormal silencing of genes 42, 45, 46.

Reader‐based stimulation of enzymatic activity may be a widely employed strategy to control histone demethylase activity, as it was recently shown that a PHD domain in KDM5 enzymes can bind to unmodified H3K4 and stimulate the activity of the enzyme towards H3K4me 47, 48. Furthermore, a second PHD domain in these enzymes binds preferentially to H3K4me histones, suggesting that KDM5 proteins recognize both the substrate and the product of their demethylation reaction 49. The functional relevance of this interplay remains to be carefully examined *in vivo*. Nevertheless, these observations suggest there may be a concerted drive for histone demethylases to encode domains that can read the chromatin modification landscape to control histone demethylation.

### Binding DNA and interpreting the genetic code

Histone demethylases make a series of complex interactions with their histone substrates, but emerging evidence indicates that they can also directly interact with DNA. These interactions with DNA can profoundly affect histone demethylase activity and targeting. For example, recent *in vitro* binding studies have shown that KDM1A interacts non‐specifically with DNA, suggesting a DNA scanning mechanism might help to identify target substrates *in vivo* 50. Furthermore, the interaction between KDM1A and extra‐nucleosomal DNA stimulates its histone demethylase activity *in vitro*, indicating that it also functions to activate the enzyme 51.

DNA binding may constitute a more widespread and underappreciated mechanism by which histone demethylases identify target regions in the genome and catalyse demethylation. This is supported by the observation that KDM2 demethylases encode a Zn‐finger CxxC domain that specifically recognizes non‐methylated CpG dinucleotides, targeting these enzymes to regions of the genome associated with gene promoters, called CpG islands (Fig 1A) 52. Here, the KDM2 enzymes play an important role in removing H3K36me1/2, a histone modification associated with transcriptional repression, suggesting the KDM2 enzymes protect CpG island regulatory elements from this repressive modification 53, 54. Interestingly, a number of other histone demethylases encode potential DNA‐binding domains, including several distinct classes of Zn‐finger and ARID DNA‐binding domains 35, 55. It will be interesting to examine in more detail how these potential DNA‐binding activities are integrated with histone reader domains to identify target sites in the genome and to regulate the activity of histone demethylases.

### Teaming up with others to regulate and alter activity

Although histone demethylases are often large multi‐domain proteins with inherent capacity to recognize target chromatin, detailed proteomic studies have revealed that many of these enzymes assemble into larger protein complexes that further regulate or even dramatically change their substrate specificity. This is exemplified by KDM1A, which was originally identified as an H3K4me1/2 demethylase that forms a stable component of the CoREST protein complex 56, 57. CoREST is a large molecular machine that also contains histone deacetylase activity and, via a combination of these activities, contributes to transcriptional repression 58, 59. However, early reports also suggested that KDM1A could form an alternative complex with the androgen receptor (AR), playing a role in gene activation of AR target genes via the removal of repressive H3K9me1/2 modifications 60. It was proposed that this switch in substrate specificity was partly mediated by histone H3T6 phosphorylation during AR‐mediated gene activation, which prevented H3K4me1/2 demethylation by KDM1A 61. However, at the biochemical level, it has remained enigmatic how KDM1A could so dramatically change its target substrate specificity. A potential explanation for these puzzling observations came recently with the discovery that a splice variant of KDM1A, called KDM1A+8a, interacts with a protein called SVIL. This interaction activates H3K9me1/2 demethylase activity in KDM1A+8a and, in this context, KDM1A functions as an activator of transcription 62. While this study did not directly test whether this KDM1A variant could account for the alteration in KDM1A substrate specificity upon interaction with the AR, it is interesting to note that SVIL has previously been shown to bind to the AR 63. Together, these observations suggest that subtle alterations in the amino acid sequence of histone demethylases and interaction with defined protein partners can profoundly affect substrate specificity and activity.

### Hijacking readers to recognize substrates

As more histone demethylase protein complexes are characterized, it is becoming clear that their capacity to interact with histones via reader domains may be a central mechanism employed to identify substrates and catalyse demethylation, even when the demethylase does not itself encode a reader domain. For example, the H3K36‐specific histone demethylase NO66, which lacks histone reader domains, interacts with PHF19. PHF19 can bind to H3K36me3 through its Tudor domain, recruiting NO66 to substrates on chromatin 64. Furthermore, histone demethylases often form protein complexes with multiple reader domains. For example, the vertebrate KDM5A/JARID1A/RBP2 and KDM5B/JARID1B/PLU1 proteins encode functional PHD domains 35, 49, but also form part of a larger SIN3 histone deacetylase co‐repressor complex 65, 66, 67. The SIN3 complex contains proteins with WD‐40 repeats, which bind unmodified histones, and a chromodomain‐containing protein, MRG‐15, which binds to H3K36me3 68. Together, these observations suggest that histone demethylase complexes likely exploit multiple reader domains and combinatorial interactions with chromatin substrates to achieve appropriate targeting and activity *in vivo* (Fig 1A).

### Sequence‐specific targeting through DNA‐binding factors and non‐coding RNAs

The activity of histone demethylases and their targeting to chromatin substrates appears to significantly rely on reading histone modification state and, in some instances, generic DNA‐binding activities. In contrast, there is only a limited number of examples in which sequence‐specific DNA‐binding transcription factors have been demonstrated experimentally to directly target histone demethylases to chromatin (Fig 1B) 38, 69, 70, 71, 72. Some of these involve the KDM1 histone demethylases, often in conjunction with hormone‐dependent gene activation 60, 61, 73, 74, 75. Interestingly however, when the occupancy of histone demethylases and their proposed transcription factor targeting molecules have been compared at the genome‐scale, the overlap is often modest. For example, KDM5C/JARID1C/SMCX interacts biochemically with c‐MYC in mouse ES cells and KDM5C is enriched at c‐MYC binding sites 71. However, the majority of KDM5C‐bound regions are not occupied by c‐MYC and, similarly, a large proportion of c‐MYC‐bound sites do not show enrichment for KDM5C 71. This suggests that physical interaction between KDM5C and c‐MYC does not broadly define KDM5C occupancy on chromatin. In fact, some recent work has provided evidence that histone demethylases may actually function upstream of transcription factors to create the appropriate chromatin environment for DNA binding 76, 77.

Additional attempts to identify sequence‐specific histone demethylase targeting determinants have suggested that, in some instances, this may rely on interaction with long non‐coding RNAs (lncRNAs) (Fig 1B). For example, the KDM1A/CoREST complex can interact with the lncRNA HOTAIR, recruiting the demethylase complex to target sites and creating a repressed chromatin state 78. Similarly, an RNA‐dependent targeting mechanism has also been proposed to target H3K9me3 demethylase KDM4D/JMJD2D/JHDM3D 79. It will be interesting to understand whether lncRNAs contribute more widely to histone demethylase targeting *in vivo*.

Given that there are currently limited numbers of specific examples where transcription factors or non‐coding RNA solely define the occupancy of histone demethylases on chromatin, more generic DNA‐binding activities or histone reading domains may predominate in achieving histone demethylase targeting. Alternatively, combinatorial interactions that rely on reader domains and generic or site‐specific targeting activities may be exploited to regulate and achieve more complex chromatin‐binding patterns and functionality. Clearly, a challenge for future work remains to understand at the molecular level the determinants that drive histone demethylase chromatin‐binding patterns *in vivo*.

### Regulating histone demethylase activity through post‐translational modification

A wide range of mechanisms appear to have evolved to guide histone demethylases to their appropriate substrates on chromatin. In addition, there is an emerging body of evidence to suggest that post‐translational modification of the enzymes themselves is exploited to regulate their levels and activity. For example, the H3K9me2 demethylase KDM7C/PHF2/JHDM1E shows no apparent activity *in vitro* but becomes enzymatically active upon phosphorylation by PKA 80. Phosphorylation‐dependent activation of KDM7C stimulates its interaction with the DNA‐binding protein ARID5B, leading to the recruitment of the demethylase complex to chromatin, presumably through the generic DNA‐binding activity of ARID5B 80. Similarly, phosphorylation by Cyclin E‐CDK2 stimulates the H3K9me1/2 demethylase activity of the related protein KDM7B and this plays a role in the regulation of gene expression during cell cycle progression 81. In addition to these specific examples where post‐translational modifications control enzymatic activity, ubiquitylation and proteasomal degradation are emerging as key determinants in regulating the levels of histone demethylases. For example, multiple studies have demonstrated that histone demethylases are substrates of SCF E3 ligase complexes and can be polyubiquitylated and targeted for proteasomal degradation 82, 83, 84, 85. This appears to be particularity important for regulating the balance of histone demethylase protein levels to ensure that they function at appropriate stages during development.

## Regulating gene expression and resetting transcriptional networks

**Figure 2:**
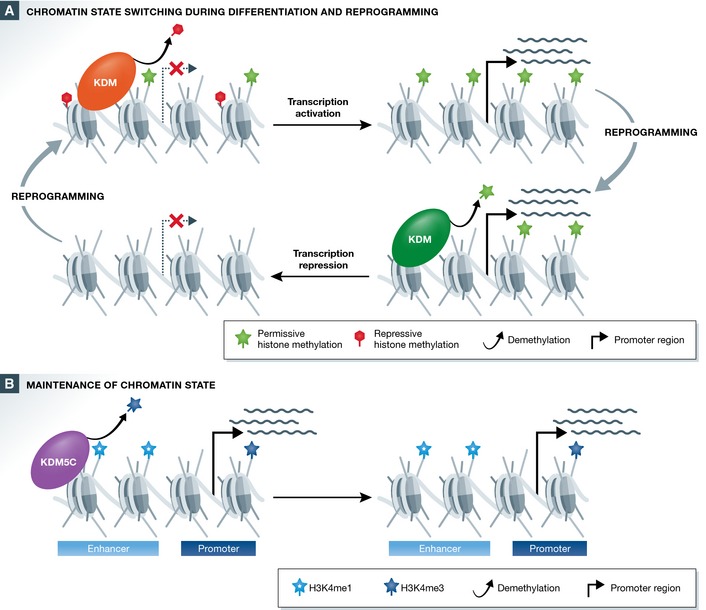
Histone demethylases shape chromatin architecture at gene regulatory elements to regulate gene expression (A) Demethylases actively remove histone methylation to establish new chromatin environments at gene regulatory elements. Removal of repressive modifications, such as H3K27me2/3, helps to create transcriptionally permissive chromatin (top), while removal of transcriptionally permissive modifications, such as H3K4me3, contributes to the formation of more repressive chromatin states (bottom). These processes appear to be particularly important in achieving new gene expression programs during lineage commitment and cellular reprogramming. (B) Histone demethylases play a key role in the maintenance of established chromatin states by preventing the spurious accumulation of alternative histone methylation states. For example, the H3K4me2/3 demethylase KDM5C contributes to the maintenance of enhancer identity by maintaining local H3K4me1 levels.

Some of the very earliest descriptions of histone modifications noted their conspicuous relationship with transcriptional activity 86, and since then, it has become clear that chromatin modifications, including histone lysine methylation, are involved in regulating gene expression. Not surprisingly, since the discovery of histone lysine demethylases, it has emerged that they contribute significantly to the specification of transcriptionally active chromatin states, transcriptional repression and cellular reprogramming events (Fig 2) 8, 10. A series of recent advances have begun to shed light on how histone demethylases contribute to these processes at a molecular level and during development.

### H3K27 demethylases contribute to the establishment of a transcriptionally permissive chromatin environment during lineage commitment

As described above, the KMD2 histone demethylases are constitutively recruited to promoter‐associated CpG islands to counteract repressive H3K36me1/2 54. This may function as a way of demarcating these regions as transcriptionally permissive. In contrast to these more generic targeting mechanisms, histone demethylases also play key roles in actively removing repressive marks from specific gene promoters during the transition from a repressed to a transcriptionally activated state 60, 62, 73, 87. This is most evident at genes required for cell lineage commitment, which are silent in embryonic and other stem cell types and occupied by the polycomb repressive complex 2 (PRC2), which places repressive H3K27me. It is thought that polycomb group proteins function at these genes to maintain their silent state and protect cell identity 88. As cells differentiate, they must acquire new transcriptional programs and do so through the activation of genes normally repressed by the polycomb system in the progenitor cell. Several *in vitro* differentiation studies have revealed an acute requirement for the KDM6 H3K27me2/3 demethylases, KDM6A/UTX and KDM6B/JMJD3, in overcoming this repressive chromatin state to achieve normal gene expression during lineage commitment 87, 89, 90, 91, 92, 93, 94. In contrast to the generic targeting mechanisms employed by the KDM2 demethylases, the KDM6A and KDM6B enzymes appear to be actively guided to specific enhancers and promoters through the function of lineage‐specific transcription factors that activate these genes 93, 95, 96, 97.

Interestingly, KDM6 recruitment to activate genes during cell lineage commitment appears to function as part of an intricate chromatin‐based cascade to initiate and then maintain gene expression. This occurs initially through the interaction of KDM6 proteins with the MLL H3K4 methyltransferase complex, leading to removal of H3K27me2/3 and deposition of H3K4me during gene activation 98, 99, 100. Following gene induction, KDM6 proteins play a second and equally important role in promoting transcription elongation. They achieve this through forming a complex with factors bound to the elongating form of RNA Pol II, including the H3K36 methyltransferase SETD2 and the elongation factor SPT6 101, 102. As transcription proceeds, KDM6 enzymes travel with the polymerase and spread into the coding regions of genes, removing H3K27me2/3 and contributing to efficient migration of RNA Pol II 93, 101, 102, 103, 104.

Although cell culture model systems have indicated an important role for the KDM6 demethylases in creating normal gene expression programs during cell lineage commitment *in vitro*, how essential their activities are for cell fate transitions during early development *in vivo* remains less clear. For example, male mice lacking both KDM6A and KDM6B survive to term and display no major increases in global H3K27me2/3. Furthermore, when double null ES cells are derived from these animals and induced to differentiate with retinoic acid, newly activated genes lose repressive H3K27me2/3 and are induced appropriately 105. In contrast, the phenotype of female KDM6A knockout mice is much more severe and they fail to undergo normal embryonic development 106, 107, 108. These sex‐specific differences in phenotype may be a result of partial compensation for loss of the X‐chromosome‐encoded KDM6A/UTX protein by the Y‐chromosome‐encoded KDM6C/UTY protein in males. Although preliminary bioinformatic and biochemical analyses of KDM6C had predicted that it was catalytically inactive due to an amino acid substitution in the JmjC domain 109, 110, simultaneous depletion of KDM6A and KDM6C in male mouse embryos phenocopies the early embryonic lethality observed in KDM6A null females 111. Based on the presumption that KDM6C is catalytically inactive, its capacity to compensate for KDM6A during development has largely been attributed to demethylase‐independent functions 111, 112. However, recent structural and biochemical evidence has demonstrated, at least *in vitro*, that KDM6C can demethylate H3K27me, albeit less efficiently than KDM6A 113. This suggests that loss of demethylase activity may indeed underpin the developmental defects observed in KDM6A/B null mice.

Together, these observations functionally implicate KDM6 proteins in early mouse development and in other cell lineage commitment models, perhaps through regulation of transcription. Nevertheless, whether KDM6 involvement in these processes relies on histone demethylation still remains unclear as most of these studies have relied on complete gene knockdown or deletion approaches. Interestingly, the *C. elegans* KDM6A orthologue, UTX‐1, is essential for worm development. However, the lethality appears to be independent of demethylase activity, with UTX‐1 instead primarily being required for formation of the UTX‐1/SET16 H3K4 methyltransferase complex 114. In contrast, studies in zebrafish have demonstrated that the demethylase activity of KDM6A is required for normal development as a catalytically deficient KDM6A protein is unable to rescue the defects in KDM6A‐depleted embryos 110. Therefore, it remains to be carefully addressed whether an inability to demethylate histones is sufficient to drive the observe phenotypes in mouse KDM6 models. New precisely engineered mouse strains, in which the catalytic domains of KDM6 enzymes are subtly mutated to abrogate catalytic activity, yet leave the remainder of the protein intact and capable of interacting with protein partners, are required. This will also provide the opportunity to examine *in vivo* the role of demethylase activity in cell lineage commitment.

### H3K4 demethylases control gene regulatory element identity and function

The process of active transcription is intimately coupled with the deposition of H3K4 methylation at gene promoters, which is thought to contribute to the activation and maintenance of gene expression 115. Based on the relationship between H3K4me and gene promoters, it is not surprising that links between KDM1A, the first identified demethylase enzyme, and the repression of gene transcription were originally identified 56, 57. Subsequently, KDM5 demethylases were discovered and shown to act at gene promoters to maintain low levels of H3K4me 69, 116, 117, 118, 119, 120, 121, 122.

Detailed genome‐wide examination of KDM1 and KDM5 protein occupancy on chromatin has provided a series of new and interesting observations that suggest that H3K4 demethylases also play critical roles in shaping H3K4me at distal gene regulatory elements, including enhancers, which are typically enriched for H3K4me1 123, 124, 125. For example, KDM1A occupies promoters and enhancers of active genes in mouse ES cells. Despite being associated with gene promoters, the loss of KDM1A in mouse ES cells does not cause major defects in the pluripotency‐associated transcriptional program nor a loss of normal cell identity 126, 127, 128. This argues that KDM1A and its demethylase activity do not profoundly affect the maintenance of normal gene expression networks in these cells. However, KDM1A activity becomes essential during differentiation, where it is required to efficiently repress ES cell specific gene expression programs during lineage commitment. This appears to rely on KDM1A removing H3K4me1 from enhancers, effectively decommissioning these regulatory elements and driving efficient transcriptional silencing of pluripotency genes during normal cellular differentiation 126, 129. In the absence of KDM1A, H3K4me1 persists at pluripotency gene‐associated enhancers and the associated genes remain partially transcribed 126. In keeping with these molecular defects observed in mouse ES cells, deletion of KMD1A in the developing embryo results in misregulation of key developmental genes and KDM1A null embryos fail to develop past embryonic day 5.5, displaying gastrulation defects 127, 128, 130.

In contrast, the KDM5 H3K4 demethylases appear to be required to maintain, rather than limit, enhancer function. Like KDM1A, KDM5C binds to gene promoters and enhancers 71. At promoters, it negatively regulates transcription by removing H3K4me2/3. However, at enhancers, it stimulates gene activity by removing spurious H3K4me3/2 modifications and maintaining enhancer‐associated H3K4me1 (Fig 2B) 71. This enhancer maintaining activity may be shared amongst KDM5 enzymes, as KDM5B also appears to restrict the spreading of H3K4me from enhancers, contributing to normal enhancer activity 131. Together, these observations suggest that KDM5 demethylases, either independently or in a partially redundant fashion, play a key role in specifying defined H3K4me states at enhancers and core promoters to promote normal gene regulation.

The distinct roles that KDM1A and KDM5 demethylases play in regulating enhancer function may in part be explained by their inherent H3K4 methylation state specificities. KDM1 enzymes can actively remove H3K4me1 and me2, whereas the KDM5 enzymes are limited to removing H3K4me2 and me3. If H3K4me1 is required for enhancer identity and function as has been proposed, it seems logical that KDM1 enzymes could support decommissioning of these elements through removal of H3K4me1 from enhancers at defined times during development. However, if transcription leads to spurious H3K4me2/3 at the enhancers of active genes, the KDM5 demethylases could constantly counteract higher methylation states to reinstate H3K4me1 and stereotypical enhancer identity (Fig 2B). These cell culture‐based observations highlight a more complex relationship between histone H3K4me demethylation and transcription regulation than was initially anticipated and suggests that methylation state specificity could underpin the differing activity of H3K4 demethylases at gene promoters and enhancers. Nevertheless, understanding whether these systems play an important role in supporting dynamic gene regulation during animal development remains an important question for future studies.

### Reprogramming the germline

Histone demethylases appear to play central roles in setting up gene expression programs during development. However, recent observations also support an interesting role for these enzymes in epigenetic reprogramming in the germline to produce gamete specific epigenomes. For example, deletion of the *C. elegans* KDM1A orthologue Spr‐5 results in progressive sterility over many generations, a process that is accompanied by the transgenerational accumulation of H3K4me2 and decreases in H3K9me3 132, 133. The balance between H3K4me2 and H3K9me3 is mediated by the function of several methyltransferases and demethylases, which can act to either suppress or enhance the observed transgenerational phenotype 134, 135. In addition, Spr‐5 cooperates with the histone remodeller LET‐418/Mi2 to maintain the germline state, counteracting H3K4 methylation and limiting unscheduled somatic differentiation 136. This suggests that Spr‐5, as part of a network of chromatin modifiers, is critical in regulating the balance between permissive H3K4 methylation and repressive H3K9 methylation during epigenetic reprogramming in gametogenesis.

Histone demethylases could potentially play a similarly important role in reprogramming chromatin states in the mouse. Indeed, during primordial germ cell specification, there is genome‐wide erasure of H3K9me2 as part of a global resetting of the epigenome; however, it remains to be examined whether this relies on histone demethylase activity 137, 138, 139. Several histone demethylases, including KDM1B, KDM3A/JMJD1A/JHDM2A and KDM3C/JMJD1C, exhibit sex‐ and stage‐specific expression patterns in the germline that correspond to changes in chromatin architecture 10, 140, 141, 142, 143. A series of knockout studies have demonstrated that KDM3A and KDM3C play essential roles in male gametogenesis, with their depletion severely affecting the formation of functional gametes and fertility 141, 142, 143. It is tempting to speculate that these enzymes may function to reset global histone methylation states in the germline in order to prevent their transgenerational transmission, as has been proposed in *C. elegans*. However, this is unlikely to be the case in the mouse male germline where the majority of histone is removed and replaced with protamine during sperm formation 144. In fact, during spermatogenesis, KDM3A appears to function to remove repressive H3K9me from the promoters of the transition nuclear protein Tnp1 and protamine Prm1 genes, supporting their expression in post‐meiotic male germ cells 142. TNP1 and PRM1 are then involved in histone replacement and sperm maturation 144. By regulating the expression of these specific genes, KDM3A indirectly results in a dramatic reconfiguration of sperm chromatin through a mechanism that does not rely on global changes of H3K9me. It will be interesting to understand whether other histone demethylases function more directly to reset epigenetic states in female gamete formation where maternal histones are not replaced by protamines.

It is clear that histone demethylases contribute to normal germline formation in some animals and it will be important to determine how directly this relies on removal of histone methylation and resetting of epigenetic states. Nevertheless, recent findings in plants demonstrate that histone demethylases also play an important role in the removal of histone methylation during plant gametogenesis, suggesting many phyla may exploit these enzymes to reset the epigenetic landscape prior to passing chromatin‐based information on to subsequent generations 145.

### Coercing cells to take on alternative cell fates

In addition to their proposed involvement in reshaping chromatin states during gamete formation and early development, histone demethylases have also been identified as key determinants in alternative reprogramming paradigms. This is evident in a naturally occurring transdifferentiation phenomenon that occurs during *C. elegans* larval development, where the epigenome of a single rectal epithelial cell is changed so that it can transform into a motor neuron 146. Using genetic screens to identify factors that contribute to or inhibit this process, it has become clear that normal transdifferentiation relies on the H3K4 methyltransferase SET‐1 and the H3K27me demethylase JMJD‐3.1. Through interactions with transcription factors, SET‐1 and JMJD‐3.1 are recruited to promoters of neuronal genes during transdifferentiation. Here, SET‐1 is thought to play a role in poising neuronal genes for activation and JMJD‐3.1 to subsequently remove repressive H3K27me to drive gene activation and efficient transdifferentiation. These activities reshape the chromatin landscape during this natural reprogramming event and help to effect gene expression programs that are required to achieve the motor neuron cell fate 146.

An understanding of the genetic determinants that support mammalian ES cell specification and maintenance led to the discovery that the introduction of certain DNA‐binding transcription factors into somatic cells under defined culture conditions could drive cellular reprogramming to an induced pluripotent stem (iPS) cell state 147. This revolutionary technique has provided new prospects for personalized medicine. However, achieving the iPS cell state is inefficient, suggesting that barriers, including the chromatin state of a somatic cell, may limit *in vitro* reprogramming. In studying this process, it has become clear that the mammalian H3K27 demethylase KDM6A is required for the active removal of H3K27me to achieve establishment of the pluripotent ground state 148. In addition, H3K9me3 was identified as a critical epigenetic barrier to reprogramming via somatic cell nuclear transfer (SCNT), and ectopic expression of the H3K9me3 demethylase KDM4D greatly improves SCNT efficiency 149, 150. Several other histone demethylases have also been found to be required for efficient reprogramming 151, 152, 153. Interestingly, the action of some histone demethylases seems to impair the reprogramming process 154, in keeping with the idea that histone demethylases likely play important roles in maintaining chromatin states in addition to establishing new ones (Fig 2B). An exciting extension of this work has shown that small molecules that counteract the activity of histone demethylases can be used to improve reprogramming efficiency 155, 156. Further studies are required to improve our understanding of the molecular mechanisms by which these histone demethylases regulate reprogramming.

### Maintaining epigenetic stability

Histone demethylases have a clear function in resetting chromatin states in the germline and other reprogramming paradigms, but studies in fission yeast suggest that histone demethylation can also function to fine‐tune how chromatin states are epigenetically transmitted to daughter cells. In fission yeast, H3K9me is targeted to centromeres by the RNAi system 157. H3K9me then spreads from these initiation sites over large distances by a copying mechanism that relies on a reader protein that binds H3K9me to recruit more methyltransferase 157, 158, 159. Given this copying mechanism, it was proposed that H3K9me chromatin domains may be epigenetically transmitted following DNA replication, with modified histones being segregated to newly replicated chromatin and sustaining initiator‐independent copying of this chromatin modification state. Surprisingly, recent studies exploiting a regulatable tethering system that allows the controlled initiation of broad domains of H3K9me at an ectopic site away from centromeres revealed that, following removal of the initiator, there was a rapid and active removal of H3K9me 160, 161. This was unexpected, as no histone H3K9 demethylase had been characterized in fission yeast. However, removal of Epe1, a JmjC domain‐containing protein, resulted in a remarkable stabilization of these ectopic H3K9me chromatin domains, allowing them to be stably transmitted across mitosis and meiosis 160, 161, 162. Demethylase activity had not been previously detected for Epe1 *in vitro* 7, 163. However, mutation of residues in Epe1 that correspond to cofactor binding sites in other active histone demethylases resulted in epigenetic stabilization of H3K9me, suggesting that Epe1 may demethylate H3K9me 160, 161. These observations are in agreement with previous results demonstrating a role for Epe1 in regulating H3K9me spreading at natural centromeres and fine‐tuning this epigenetic state to maintain normal chromosome segregation 164, 165, 166. Interestingly, Epe1 encodes a tyrosine in its active site that in vertebrate KDM7C has been shown to act as a phosphorylation‐dependent switch to activate the enzymatic activity of KDM7C 80, 167. It is tempting to speculate that Epe1 may also require this tyrosine to be phosphorylated in a regulated manner to efficiently catalyse H3K9 demethylation, and this could potentially account for the lack of demethylase activity in recombinantly produced Epe1 7, 163. Nevertheless, these new studies highlight a completely new role for histone demethylases that is distinct from simple epigenetic reprograming and suggests that histone demethylases may function to limit or control the spreading or persistence of epigenetic states. It will be interesting to examine the extent to which histone demethylase systems fine‐tune epigenetically transmitted chromatin states in higher eukaryotes.

## Histone demethylases as emerging players in regulation of DNA replication and cell division

**Figure 3:**
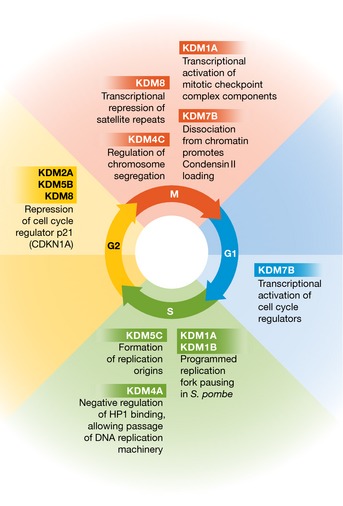
Histone demethylation is an integrated part of the cell cycle Several histone demethylases play important roles at defined stages to support normal cell cycle associated processes. For example, they contribute to the establishment of chromatin states that are required for the expression of important cell cycle regulators, DNA replication, segregation of chromosomes, and genomic stability during cell division. Misregulation of these histone demethylases, or their activity, often causes cell cycle arrest and may also lead to genomic instability in cancer.

In addition to the central roles that histone lysine demethylases play in gene regulation, cell fate decisions and reprogramming, it has recently emerged that these enzymes are also involved in fundamental molecular processes that underpin DNA replication, cell cycle dynamics and cell division (Fig 3).

### Forming origins and DNA replication

The initiation of DNA replication and the copying of genetic information is a highly regulated and precisely controlled process. Establishing the correct chromatin environment is essential for proper formation of replication origins and replication itself 168, 169, 170. Interestingly, recent studies have implicated histone demethylases in several aspects of DNA replication. For example, the H3K4me3 demethylase KDM5C appears to play an important role in forming origins and initiating replication at actively transcribed early‐replicating genes 171. This relies on an elevated expression of KDM5C during early S phase where it functions to actively remove H3K4me3 from replication origins, promoting the formation of the pre‐initiation complex and driving occupancy of PCNA. In the absence of KDM5C, or its demethylase activity, H3K4me3 persists at these sites and early origins fail to efficiently initiate replication, leading to cell cycle arrest 171, 172. It is still unclear precisely how removal of H3K4me3 is involved in this process; however, several proteins in the origin of replication complex are known to encode chromatin reader domains 173. Perhaps components of the origin of replication complex or other replication‐associated factors are responsive to the modification state of H3K4.

Once replication has been initiated, the process of ongoing replication is also regulated by the activity of histone demethylases. The levels of the H3K9me3 demethylase KDM4A/JMJD2A/JHDM3A are elevated at S phase, coincident with loss of H3K9me3 and an increase in H3K9me1/2 during replication 174, 175. H3K9me3 in chromatin is normally bound by the chromodomain‐containing protein HP1γ, which contributes to the formation of condensed heterochromatic structures 176. During S phase, KDM4A demethylase activity counteracts HP1γ binding at heterochromatic regions, creating more accessible chromatin required for passage of the DNA replication machinery 174. This system appears to be tightly controlled through the cell cycle by regulating KDM4A protein levels which is required for accurate replication timing 83, 174. KDM4A‐dependent effects on DNA replication are observed in mammalian cells and the model organism *C. elegans*, suggesting that this is an evolutionarily conserved function of the enzyme 174. In keeping with a role for KDM4A activity in controlling DNA replication, overexpression of KDM4A leads to genomic instability in a demethylase‐dependent manner, through driving re‐replication and site‐specific copy gain in genomic regions implicated in cancer 177.

As we begin to understand more about the function of the histone demethylases, it seems likely that they have more widespread, conserved and even co‐opted functions in the regulation of DNA replication. This is supported by observations in *S. pombe* demonstrating that KDM1A and KDM1B contribute to programmed replication fork pausing that promotes imprinting and mating‐type switching 178. Together, these observations suggest that there is likely an underappreciated role for histone demethylases in regulating the processes that initiate and regulate accurate replication of the genome.

### Cell cycle transitions and organizing chromosomes

Control of cell cycle timing and dynamics is essential for proper cell division and recent work has demonstrated that histone demethylases play several distinct roles in controlling normal cell division (Fig 3) 10. One specific way this is achieved is through their capacity to directly regulate the expression of genes required for normal cell cycle progression 72, 81, 179, 180, 181, 182. This is exemplified by the demethylase KDM7B, which binds to the promoters of several key cell cycle regulators, including E2F1 target genes, and is required for their transcriptional activation by removing the repressive H3K9me1/2 and H4K20me1 179. In keeping with this role, KDM7B protein levels and its binding to chromatin are highly regulated during the cell cycle and this appears to play important roles in the G1/S and G2/M transitions 81, 179, 180. Similarly, KDM1A positively regulates the expression of MAD2 and BUBR1, which are part of the mitotic checkpoint complex and are required for proper chromosome segregation during mitosis 181. Transcriptional regulation by histone demethylases also ensures genomic stability during cell division 183, 184, 185 possibly by removing modifications associated with transcriptionally permissive chromatin states during mitosis 184, 186. For example, KDM8/JMJD5 is involved in repression of transcription at non‐coding satellite repeat regions, possibly by removal of H3K36me2. In the absence of KDM8 activity, elevated H3K36me leads to defective spindle formation and causes abnormal cell division and genomic instability 184. However, the mechanism by which KDM8 regulates H3K36me remains contentious as other studies failed to observe histone demethylase activity for KDM8 and, instead, suggest that KDM8 may act as a protein hydroxylase 187, 188, 189.

Interestingly, during cell cycle transitions, histone demethylases can also function independently of their effects on gene transcription. As cells enter into prophase of mitosis, they need to deposit H4K20me1 on chromatin in order to load Condensin II, a structural protein complex required for chromosome condensation 179, 190. As chromatin‐bound KDM7B would normally demethylate H4K20me1, its removal from chromatin is required to stabilize H4K20me1 and promote this transition. The cell achieves this through CDK1/cyclin B‐dependant phosphorylation of KDM7B, which then leads to KDM7B dissociation from chromatin in prophase 179. Although this dynamic engagement between KDM7B and chromatin is in fitting with its functions during the cell cycle, other histone demethylases appear to support normal chromosome segregation through alternative mechanisms. KDM4C/JMJD2C/JHDM3C remains associated with chromosomes throughout mitosis and is proposed to maintain low levels of H3K9me and regulate chromosome segregation 183. However, deletion of KDM4C in mouse does not appear to overtly affect development, physiology or reproduction, suggesting that some of the effects observed in cell culture may not completely reflect an essential requirement *in vivo* 191. Moving forward, a better understanding of how histone demethylases are involved in cell cycle progression and cell division in animals will be essential, given that misregulation of these enzymes appears to play roles in proliferation and cell division in cancer.

## Protecting the genome by regulating the DNA damage response

In order to protect the integrity of genetic information, living organisms exploit highly specialized systems to sense and repair DNA damage. In eukaryotes, these systems have evolved to use histone post‐translation modifications as key regulators of the DNA damage response 192. Fittingly, there appears to be a concerted drive to regulate how histone demethylases engage with chromatin and also to precisely control the levels of these proteins during damage sensing and repair.

### Modifying demethylases to alter chromatin binding and regulate histone methylation during the DNA damage response

Like a host of other chromatin‐modifying enzymes, KDM4B/JMJD2B/JHDM3B and KDM4D are specifically recruited to sites of DNA damage. This relies on their post‐translational poly‐ADP ribosylation by PARP1, a key signalling event that drives early cellular responses to DNA damage 193, 194. Experimental depletion of KDM4D impairs the formation of DNA damage‐induced RAD51 and 53BP1 foci and this inhibits double‐strand break repair through homology‐directed repair and non‐homologous end joining 193. The contribution of KDM4 proteins to the repair process relies on their demethylase activity, and a rapid decrease in H3K9me is observed in response to DNA damage, suggesting that these effects are mediated through chromatin 193, 194. Interestingly, there may be a more concerted PARP1‐dependent drive to recruit demethylase activity to sites of DNA damage. PARylated KDM5B is recruited to macroH2A1.1 at double‐stranded DNA breaks, where its demethylase activity is required to nucleate Ku70 and BRCA1 and effect non‐homologous end joining and homology‐directed repair 195. These studies suggest that PARylation may be an important driver of the histone demethylase response to DNA damage. However, the molecular mechanisms that integrate PARylation with the recruitment of these demethylase enzymes to sites of DNA damage and mechanistically how the removal of histone methylation contributes to DNA repair process remain poorly defined and interesting areas for future work.

Other post‐translational modifications also appear to control how histone demethylases respond to DNA damage. During DNA damage, KDM1A is phosphorylated by CK2, allowing it to interact with RNF168 which then recruits KDM1A to sites of DNA damage. Once bound KDM1A removes H3K4me2 and is required for normal 53BP1 recruitment and DNA repair 196, 197. In addition, the KDM2A H3K36 demethylase is phosphorylated by ATM kinase in response to double‐strand breaks and, instead of recruiting KDM2A to break sites, phosphorylation abrogates its chromatin‐binding activity. This is proposed to protect H3K36me2 at damage sites, which helps to recruit the MRE11 complex to efficiently repair double‐stranded breaks 198, 199. Overexpression of KDM2A, but not a phosphomimetic mutant, leads to decreased H3K36me2, inefficient double‐strand break repair and reduced cell survival 198, 199. In addition to phosphorylation, the SUMOylation of histone demethylases has recently emerged as a novel regulator of targeting during the DNA damage response. KDM5C is SUMOylated in response to DNA damage and this causes an increase in its chromatin occupancy where it removes transcriptionally permissive H3K4me3, which is proposed to contribute to transcription inhibition prior to DNA repair 200. Understanding the molecular mechanisms through which post‐translational modifications regulate the engagement of histone demethylases with chromatin remains a key challenge in elucidating how the DNA damage response exploits chromatin modification in sensing and repairing DNA damage, and also in further defining how demethylases recognize and are recruited to new chromatin substrates.

### Turning over histone demethylases in response to DNA damage

If executed correctly, sensing and then effecting DNA repair is a multistep process that by its very nature is dynamic. Therefore, it is not surprising that many of the factors involved occupy damage sites in a transient and regulated manner. In agreement with this, live cell imaging has shown that KDM1A and KDM4 recruitment to DNA damage sites occurs early in the damage response and that their occupancy is transient 193, 194, 196, 197. An understanding of the precise mechanisms that underpin these transient interactions at DNA damage sites is currently limited, but in the case of the KDM4 demethylases this may in part be driven by active protein turnover. During the DNA damage response, RNF8 and RNF168 are recruited to sites of damage and polyubiquitylate KDM4A and KDM4B, leading to their proteasomal degradation 201. This was originally proposed to act as a generic mechanism to dislodge KDM4 from chromatin at DNA damage sites, as KDM4 enzymes encode H4K20me‐binding Tudor domains that could block efficient occupancy of the damage response protein 53BP1 201, which also recognizes this modification 202, 203. Given that we now know that KDM4 enzymes are also actively targeted to sites of DNA damage 193, 194, 204, it is tempting to speculate that during the early stages of the DNA damage response, KDM4 activity counteracts H3K9me3 which might normally create chromatin structures that are inhibitory to the DNA repair process. Following demethylation, the recruitment of RNF8 and RNF168 to these sites could then evict the KDM4 enzymes to create a binding site for 53BP1. This would reconcile observations that both the recruitment and removal of KDM4 enzymes from chromatin are required for the formation of 53BP1 foci and DNA repair. It is likely that additional histone demethylases will also be subject to proteasomal control in shaping the DNA damage response, as it was recently shown that SUMOylated KDM5B is ubiquitylated by the SUMO‐specific E3 ligase RNF4, leading to the proteasomal degradation of KDM5B in response to DNA damage 200. Together, these new insights are beginning to reveal how histone demethylases help to shape the DNA damage response and suggest that they play important roles in maintaining genomic integrity. As these studies are still in their infancy, it remains a future challenge to understand how histone demethylation contributes to the repair processes at the molecular level and to determine whether misregulation of histone demethylases has direct implications for genome integrity in cancer.

## New functions that are independent of histone demethylation

**Figure 4:**
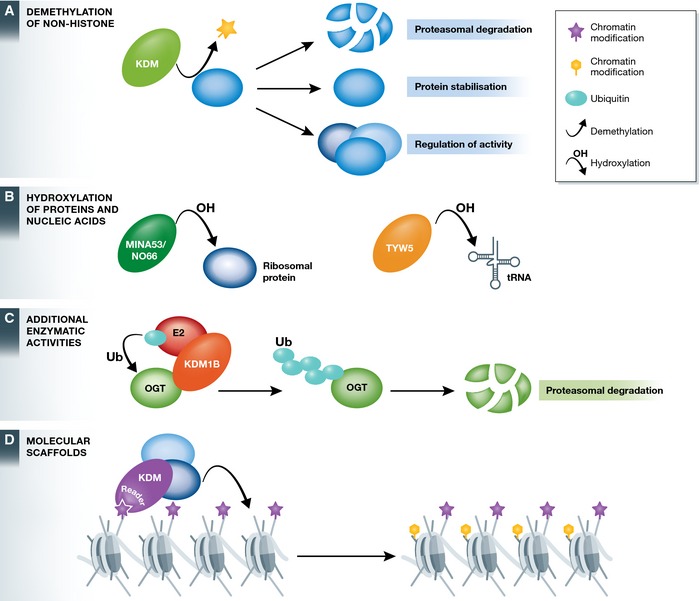
Emerging functions that are independent of histone demethylation (A) Histone demethylases have also been demonstrated to remove methyl groups from non‐histone protein substrates to regulate their abundance, stability or activity. (B) Histone demethylases function more generally as 2‐OG oxygenases, catalysing the hydroxylation of various protein and non‐protein substrates, including ribosomal proteins, transcription factors and tRNA. (C) Histone demethylases can possess alternative enzymatic activities. For example, KDM1B functions as an E3 ubiquitin ligase that ubiquitylates OGT leading to its proteasomal degradation. (D) Histone demethylases appear to function as molecular scaffolds, exploiting their chromatin‐binding capacity to recruit other proteins and chromatin remodelling activities.

As discussed above, histone demethylases contribute significantly to gene expression, chromatin organization and genomic integrity. In most cases, this has been attributed to their histone demethylase activities. However, it has more recently emerged that these proteins also have numerous activities that are distinct from histone demethylation (Fig 4), raising the question of whether their primary functions inside the cell rely on histone demethylase activity.

### Protein demethylases rather than just histone demethylases?

There are now numerous examples where demethylase enzymes with previously defined roles in histone demethylation also appear to demethylate non‐histone proteins to regulate their abundance, stability or activity (Fig 4A). For example, KDM1A can demethylate p53, blocking its function as a transcriptional activator by preventing an interaction between p53 and 53BP1 205. KMD1A also demethylates the transcription factor E2F1 in response to DNA damage. This stabilizes E2F1 and promotes apoptosis via induction of E2F1 target genes 206, 207. Interestingly, the stability of the central DNA methyltransferase, DNMT1, is also subject to a lysine methylation–demethylation cycle that is regulated by KDM1A, and, therefore, KMD1A demethylase activity indirectly controls global DNA methylation levels during development 128.

Importantly, a series of studies have expanded on these observations and shown that several JmjC domain‐containing demethylases are similarly involved in the demethylation of non‐histone substrates 80, 208, 209, 210, 211, 212, possibly justifying an argument that histone demethylases should instead be considered protein demethylases. With this in mind, recent large‐scale proteomic studies have demonstrated that a much larger fraction of non‐histone proteins are methylated than previously appreciated 213, 214. This includes a wide range of transcription factors, regulators of chromatin organization and proteins involved in many other nuclear and cytoplasmic processes 213.

In fitting with JmjC domain‐containing demethylases also functioning on cytoplasmic substrates, a new and unexpected role for KDM4A in protein synthesis has recently been identified 215, 216. KDM4A was shown to associate with the translation machinery and regulate the distribution of initiation factors on polysomes. Interestingly, depletion of KDM4A led to reduced protein synthesis. Although the mechanism by which KDM4A regulates protein translation is still unclear, it seems likely that this relies on its demethylase activity, as treatment with a small molecule inhibitor of the JmjC domain led to defects in translational initiation.

This realization that demethylases potentially play widespread roles in protein demethylation raises an important question of whether the primary biological functions currently attributed to demethylases result from histone demethylation or other uncharacterized non‐histone protein demethylase activities. A wealth of new information detailing protein methylation and the realization that histone demethylases function more broadly as protein demethylases highlight a new and emerging role for dynamic protein methylation in basic biology. Future work focussed in this area is required to understand how demethylase enzymes are involved in these processes.

### Other reactions catalysed by JmjC domain‐containing proteins

JmjC domain‐containing demethylases comprise a large family of more than 30 proteins in human. Central to their demethylase activity is an oxygenase activity that couples decarboxylation of 2‐OG with the oxidation of N‐methyl groups, leading to the spontaneous release of formaldehyde and ultimately demethylation. Therefore, the primary reaction catalysed by these enzymes is actually a hydroxylation reaction. A systematic analysis of the substrate‐selectivity of different JmjC catalytic domains *in vitro* demonstrated that these enzymes have the capacity to function more broadly as protein 2‐OG oxygenases, catalysing the removal of other N‐alkyl groups, in addition to methyl groups 189. In keeping with these alternative substrates, the JmjC domain‐containing proteins MINA53 and NO66 catalyse the histidyl hydroxylation of ribosomal proteins 217, 218 and KDM8 has been proposed to hydroxylate the transcription factor NFATc1 to promote its proteasomal degradation 187, 188. These observations are also supported by structural studies showing that this hydroxylase activity is evolutionary conserved from bacteria to humans 217, 218. Similarly, JMJD6 has been shown to catalyse the lysyl hydroxylation of the splicing factor U2AF65 and to be involved in regulation of mRNA splicing 13, 219. Interestingly, JMJD6 was also reported to specifically bind single‐stranded RNA 219, 220, raising the possibility that it may also modify RNA. In fact, another JmjC domain‐containing protein, TYW5, acts as a tRNA hydroxylase 221. Together, these findings demonstrate that the function of JmjC domain‐containing proteins may extend far beyond protein demethylation, supporting a complex series of protein and nucleic acid hydroxylation reactions that provide potentially exciting new regulatory principles in biological processes (Fig 4B).

### A demethylase with split (enzymatic) personality

In studying histone demethylases, their activity towards methylated substrates has been the main focus. As this large class of proteins is studied in more detail, new and more diverse functions are likely to emerge. This has recently been the case for KDM1B, which also appears to function as an E3 ubiquitin ligase independently of its H3K4me1/2 demethylase activity. As an E3 ligase, KDM1B targets polyubiquitylation of the O‐GlcNAc transferase OGT, which leads to its proteasomal degradation (Fig 4C) 222. OGT is often upregulated in cancer and has been previously linked to regulation of gene expression via O‐GlcNAcylation of chromatin‐binding factors 223. For example, OGT O‐GlcNAcylates and cleaves HCF‐1, which promotes HCF‐1 proteolytic maturation 224, 225. HCF‐1 is a component of SET1/MLL H3K4 methyltransferase complexes and promotes their recruitment to chromatin 226, 227, 228, 229. Therefore, OGT is both a regulator of H3K4 methyltransferase complexes and itself regulated by a H3K4 demethylase, suggesting it may play a central role in coordinating H3K4 methylation. Importantly, loss of KDM1B E3 ligase activity leads to abnormal expression of a group of oncogenes, demonstrating that KDM1B may act as a suppressor of tumorigenesis through its E3 ligase activity and effects on OGT stability 222. It will be interestingly to examine whether other histone demethylases also have enzymatic activities outside of their well‐characterized roles in hydroxylation and demethylation.

### It is not all about enzymatic activity—demethylases as molecular scaffolds

Histone demethylase proteins often encode chromatin‐binding domains and are part of large multiprotein complexes. In some instances, this allows them to recruit their associated proteins to chromatin in a manner that does not rely on demethylase or hydroxylase activity, effectively allowing them to function as molecular scaffolds that target other chromatin‐modifying activities (Fig 4D). This appears to be the case for KDM2B, which stably associates with polycomb repressive complex 1 (PRC1), an H2AK119 E3 ubiquitin ligase, and targets the complex to CpG islands via the KDM2B Zn‐finger CxxC DNA‐binding domain, without a requirement for histone demethylase activity 230, 231, 232, 233. Similarly, JARID2, another JmjC domain‐containing protein which lacks demethylase activity altogether, is required for targeting PRC2 to chromatin 234. This suggests that histone demethylases, and their inherent chromatin‐binding activities, may have been co‐opted in certain instances to drive the recruitment of proteins complexes that carry out functions that do not directly require their enzymatic activity.

Histone demethylases have also been demonstrated to contribute to chromatin organization through targeting nucleosome remodelling factors. A recent study demonstrated that KDM3A functions as a signal‐sensing scaffold linking PPARγ and the SWI/SNF chromatin remodelling complex to long‐range promoter/enhancer interactions in gene regulation 235. This scaffolding mechanism relies on phosphorylation of KDM3A by PKA during β‐adrenergic stimulation in adipocytes and is important for the activation of key thermogenic genes 235. Similarly, KDM6 demethylases have also been proposed to play a role in chromatin remodelling by linking T‐box transcription factors and SWI/SNF chromatin remodelling complexes through mechanisms that are independent of their enzymatic activity 112. These examples highlight potentially novel roles for histone demethylases proteins as molecular scaffolds that support protein and chromatin interactions.

## Conclusion and outlook

A decade on from the initial discovery of histone lysine demethylases, our understanding of how these fascinating enzymes function in cells has progressed at an immensely rapid pace. During this time, the emergence of genome‐wide technologies has allowed us to examine the function of these enzymes on chromatin with unprecedented breadth and precision. This has provided a surprisingly detailed understanding of the fundamental roles that these enzymes play in controlling gene expression, cell fate decisions during development, and the reprogramming of chromatin states. Furthermore, new functions for histone demethylases as critical regulators of other important cellular processes, including DNA replication, cell cycle dynamics and the repair of DNA damage, have been identified that clearly warrant further investigation.

Perhaps not surprisingly given their discovery as histone demethylases, these enzymes and their cellular functions have been studied within the guise of histone demethylation. However, it is now increasingly clear that these proteins also catalyse other hydroxylation reactions that regulate both protein and nucleic acid based processes. A clear challenge for the future will be to understand the primary molecular determinants that underpin the phenotypes that result from perturbing demethylase enzymes. Does this rely on histone demethylase activity, protein demethylase activity or the hydroxylation of other cellular substrates? Alternatively, are these outcomes driven independently of enzymatic activity all together? Addressing these important questions, particularly within the context of developmental transitions where these proteins appear to be of central importance, will inevitably rely on the generation of new animal models, where specific activities can be disrupted to study and define the molecular principles that underpin the function of these fascinating proteins in normal biology and, ultimately, disease.

## Conflict of interest

The authors declare that they have no conflict of interest.

**Sidebar A: In need of answers**

- How much do chromatin reader and sequence‐specific recruitment mechanisms contribute to histone demethylase target recognition and activity? Are these functions integrated?
- Is there interplay or coordination between the function of histone demethylases that have the same substrates? If so, how is this regulated during development?
- How do histone demethylases recognize sites of DNA damage and how do they contribute to DNA repair at the molecular level?
- Do the phenotypes observed in knockout animal models result from the loss of histone demethylase activity or other demethylase‐independent functions?
- Is removing methyl groups from histones the primary function of histone demethylases?
